# Beneficial Effects of a Q-ter® Based Nutritional Mixture on Functional Performance, Mitochondrial Function, and Oxidative Stress in Rats

**DOI:** 10.1371/journal.pone.0010572

**Published:** 2010-05-11

**Authors:** Jinze Xu, Arnold Y. Seo, Darya A. Vorobyeva, Christy S. Carter, Stephen D. Anton, Angela M. S. Lezza, Christiaan Leeuwenburgh

**Affiliations:** 1 Division of Biology of Aging, Genomics and Biomarkers Core of The Institute on Aging, Department of Aging and Geriatric Research, University of Florida, Gainesville, Florida, United States of America; 2 Department of Biochemistry and Molecular Biology “E. Quagliariello” University of Bari, Bari, Italy; Universidad Europea de Madrid, Spain

## Abstract

**Background:**

Mitochondrial dysfunction and oxidative stress are central mechanisms underlying the aging process and the pathogenesis of many age-related diseases. Selected antioxidants and specific combinations of nutritional compounds could target many biochemical pathways that affect both oxidative stress and mitochondrial function and, thereby, preserve or enhance physical performance.

**Methodology/Principal Findings:**

In this study, we evaluated the potential anti-aging benefits of a Q-ter® based nutritional mixture (commercially known as Eufortyn**®**) mainly containing the following compounds: terclatrated coenzyme Q_10_ (Q-ter®), creatine and a standardized ginseng extract. We found that Eufortyn**®** supplementation significantly ameliorated the age-associated decreases in grip strength and gastrocnemius subsarcolemmal mitochondria Ca^2+^ retention capacity when initiated in male Fischer344 x Brown Norway rats at 21 months, but not 29 months, of age. Moreover, the increases in muscle RNA oxidation and subsarcolemmal mitochondrial protein carbonyl levels, as well as the decline of total urine antioxidant power, which develop late in life, were mitigated by Eufortyn**®** supplementation in rats at 29 months of age.

**Conclusions/Significance:**

These data imply that Eufortyn**®** is efficacious in reducing oxidative damage, improving the age-related mitochondrial functional decline, and preserving physical performance when initiated in animals at early midlife (21 months). The efficacy varied, however, according to the age at which the supplementation was provided, as initiation in late middle age (29 months) was incapable of restoring grip strength and mitochondrial function. Therefore, the Eufortyn**®** supplementation may be particularly beneficial when initiated prior to major biological and functional declines that appear to occur with advancing age.

## Introduction

Mitochondrial dysfunction and oxidative stress are recognized as fundamental driving mechanisms of aging [1]–[3]. The aging process results in an accelerated decline of functional performance including a decline in physiological function and reduced physical activity [4]. The exact mechanisms that cause this functional decline are not fully understood; however, the mitochondrial free radical theory of aging has gained strong support [1], [5], [6]. With advancing age, oxidative damage (e.g. mtDNA mutations) accumulates in individual cells; eventually some cells reach the point at which the mitochondrial energy generation system is seriously impaired. When the impairment of mitochondrial bioenergetics occurs in a significant number of cells within a tissue, the function of the tissue is compromised and consequently contributes to age-related pathologies. Since mitochondrial dysfunction and aging are mediated by a number of biochemical pathways, interventions that target multiple pathways through combination therapies may be more efficacious than therapies that only target one pathway [7]–[9].

Nutritional based interventions are becoming more popular among consumers and specific combinations of nutritional interventions may effectively bypass electron transport chain defects, provide alternative energy sources, and act as antioxidants as well [7]–[9]. Thus, specific nutritional mixtures may have the potential to reduce oxidative stress, prevent mitochondrial functional decline, and preserve performance. Three specific compounds that have been found to affect both oxidative stress and mitochondrial function are coenzyme Q_10_ (CoQ_10_), creatine, and ginseng. These compounds may act in a synergistic manner when combined.

Coenzyme Q_10_ (CoQ_10_) has long been known for its key role in mitochondrial bioenergetics and prescribed for chronic fatigue syndrome [10], [11]. In its reduced form, it is known as ubiquinol, a potent lipophilic antioxidant [12]. CoQ_10_ is a component of the electron transport chain and is associated with inner mitochondrial complex III protein and participates in aerobic cellular respiration [13], [14]. It is also located in membranes in close proximity to the unsaturated lipid chains to act as a primary scavenger of free radicals [15]. Also it is recognized as a modulator of mitochondrial permeability transition, which is associated with the mitochondria-mediated apoptosis and cell death [16], [17]. The opening of permeability transition pores (PTP) in the inner mitochondrial membrane can lead to mitochondrial swelling and dysfunction, causing cell death [18]–[20]. CoQ_10_ appears to exert its protective effect by inhibiting the mitochondrial PTP opening [21]–[23]. Moreover, CoQ_10_ is a cofactor of mitochondrial uncoupling proteins and may also exert protective effects by activation of these proteins, leading to a reduction in mitochondrial-free radical generation [24], [25]. Protective effects of CoQ_10_ against oxidative stress and mitochondrial dysfunction have been demonstrated in several *in vitro* and *in vivo* models [21], [26]–[30]. The capability of CoQ_10_ to trigger mitochondrial function is thus grounded on sound biochemical evidence. However, its use in humans as a therapeutic agent proved unsatisfactory, most likely because of its poor solubility in water, its limited solubility in lipids, and its relatively high molecular weight (863 g/mol), all result in poor oral bioavailability [31], [32]. For example, in one study in rats it was reported that only about 2–3% of orally administered CoQ_10_ was absorbed [33]. Recently, a mechano-physical procedure called terclatration was developed, whereby CoQ_10_ is rendered highly water soluble without chemical modification of the moiety [34]. The resultant multicomposite, Q-ter®, can be shown to be 3- to 5-fold more bioavailable in humans, as compared to native CoQ_10_ [34]. Fetoni *et al*. reported that water-soluble Q-ter® shows an improved ability versus normal CoQ_10_ in preventing oxidative damage and mitochondrial dysfunction in noise-induced hearing loss in guinea pigs [31].

The two other compounds utilized in Eufortyn® are creatine and ginseng each targeting different biological pathways [35], [36]. Creatine supplementation was initially used as an ergogenic aid to increase the phosphocreatine pool within muscle to bolster athletic performance [37]–[40]. It has been also reported to act as an antioxidant against reactive species ions, e.g. superoxide anions (O•^−^_2_) and peroxynitrite (OONO^−^) [41], and enhance expression of myogenin and other myogenic regulatory factors that regulate myosin heavy chain expression as well [42], [43]. However, creatine supplementation has recently been recognized as a potential intervention to various neurodegenerative disorders targeting bioenergetic failure observed in these conditions [44], [45]. For example, creatine has been found to be useful in buffering intracellular energy stores and reducing the cellular energy deficit by improving mitochondrial function and to be effective in attenuating the age-related decline in mitochondrial function [44]. Creatine has also been shown to inhibit mitochondrial permeability transition and enhance mitochondrial function in stimulating mitochondrial energy production via creatine-stimulated respiration and the adenine nucleotide translocase [35], [46].

Moreover, ginseng is a potent antioxidant and has been extensively used to reduce oxidative damage and prevent age-related diseases [47]–[49]. Ginsenosides, the major active ingredients of ginseng, are steroidal saponins with different sugar moieties [50]. To date more than 30 types of ginsenosides have been isolated and identified [51]. In particular, ginsenoside Rb1 and Rg1 are considered to be the most effective compounds in ginseng extracts [48], [52]. Various studies provide compelling evidence that ginseng extracts possess mitochondria protective capacity by inhibiting mitochondrial swelling and preserving mitochondrial membrane integrity in rodents [36], [53], [54].

In this study, we evaluated the potential effects of a nutritional mixture mainly containing Q-ter®, creatine and a Rg1 titrated ginseng extract (the mixture is commercially known as Eufortyn®) using Fischer 344 x Brown Norway rats on health span. Specifically, we examined muscle mitochondrial permeability transition pore opening and protein carbonylation as well as muscle cellular nucleic acid oxidation and functional outcome to explore the effectiveness of Eufortyn® on anti-aging aspects.

## Materials and Methods

### Animals and experimental procedures

The study was approved by the Institutional Animal Care and Use Committee at the University of Florida. All procedures were performed in accordance with the National Institutes of Health guidelines for the care and use of laboratory animals. Forty-seven Fischer 344 x Brown Norway (F344BNF1) male rats were obtained from the National Institute of Aging colony (Indianapolis, IN) and acclimated to the animal housing room of the University of Florida for 2 weeks. The rats were housed individually in a temperature (20±2°C) and light-controlled environment (12-hour light/dark cycle) with regular rat chow and water available *ad libitum*. The treatment started after acclimation by adding Eufortyn® compounded in a highly palletable food pellet (see next detailed section on composition and supplementation). After four weeks' of treatment, grip strength was measured. During the last week of the experimental period, rats were housed in metabolic cages for urine collection. Collected urine was immediately aliquoted and stored at −80°C until analysis. Finally, rats were euthanized by rapid decapitation for collection of trunk blood and skeletal muscle after six weeks' of treatment. Body weights were measured weekly and health status was checked by a veterinarian daily.

### Composition of the nutritional mixture and supplementation pellet preparation

The commercially available nutritional mixture, Eufortyn®, supplied by Pharmaland SA, Republic of San Marino, was used (**Table 1**). The main ingredients are CoQ_10_ (5.3 mg/g of dry powder) creatine (56.7 mg/g) and ginseng (21.7 mg/g). Coenzyme Q_10_ in the mixture is present as the terclatrate, Q-ter®, in which CoQ_10_ stands in a 1∶9 ratio with a pharmaceutically inactive matrix. Q-ter® itself is a multicomposite substance, obtained through the mechano-physical procedure called terclatration, whereby CoQ_10_ is embedded in the matrix and rendered highly water soluble without chemical modifications of the moiety [34].

**Table 1:** The composition of Eufortyn® powder used for integration into animal supplementation pellets by Bio-Serv.

Ingredients	Content (mg/g)
Q-ter®	5.3
Creatine	56.7
*Ginseng panax* root extract	21.7
Nicotinamide (Vit PP)	1.7
Calcium panthotenate (Vit B5)	0.7
Riboflavin (Vit B2)	0.3
Thiamine hydrocloride (Vit B1)	0.2
Piridoxin hydrochloride (Vit B6)	0.2
Cianocobalamine (Vit B12)	0.2×10^−3^
Magnesium aspartate	40.7
Potassium aspartate	40.7
Sucrose	83.3

The Eufortyn® supplementation food pellets and control bacon flavour pellets for the animals were prepared by Bio-Serv (Frenchtown, NJ) utilizing an effective bacon flavour masking capability in a grain-based diet.

The Eufortyn® supplementation food pellets and control bacon flavour pellets for the animals were prepared by Bio-Serv (Frenchtown, NJ) utilizing an effective bacon flavour masking capability in a grain-based diet (**Table 2**). A standard dose of 5 gram pellet contained Eufortyn® ingredients of 5 mg of terclatrated CoQ_10_ (Q-ter®), 53 mg of creatine, and 20 mg of ginseng extract. Each rat received approximately one supplementation pellet (Eufortyn® or control) every day and pellet size was adjusted daily according to the body weight of individual animal to ensure that the rat received proper dose of Eufortyn® (**Table 2**). The following daily dosage: 10 mg/kg of terclatrated CoQ_10_, 106.25 mg/kg of creatine, and 40.63 mg/kg of ginseng extract were administered for six weeks.

**Table 2:** The ingredients of Eufortyn® and control pellets supplemented to rats.

Ingredients (mg/pellet)	Control	Eufortyn®
Q-ter®	0	5
Creatine	0	53
Ginseng	0	20
Protein	1055	1055
Fat	200	200
Carbohydrates	2750	2750
Fiber	200	200
Ash	335	335
Moisture	<500	<500

Each rat received approximately one supplementation pellet (Eufortyn® or control diet) daily for 6 weeks before euthanasia and pellet size was adjusted daily according to the body weight of each individual animal to ensure that a constant dose of Eufortyn® was administrated.

### Grip strength

Forelimb grip strength was measured using an automated grip strength meter (Columbus Instruments, Columbus, OH) [55]–[57]. The grip force technique has been validated in many studies assessing muscular function during drug treatment or pathologies [58], [59]. It is also an indicator of muscle strength and it correlates significantly with sarcopenia and mortality [55], [57], [60]. For this procedure, the experimenter grasped the rat by the tail and suspended it above a grip ring. After about 3 sec, the animal was gently lowered toward the grip ring and allowed to grasp the ring with its forepaws. The experimenter then quickly lowered the body to a horizontal position and tugged the tail until its grasp of the ring was broken. The mean force in grams was determined with a computerized electronic pull strain gauge fitted directly to the grasping ring, and was divided by body mass. Average measurements from three (3) successful trials were taken as the final outcome. Successful trials were defined as those in which the animal grasped the ring with both forepaws and pulled the ring without jerking. Grip strength results were expressed as total grip strength force divided by body weight (kg of force/kg body weight).

### Muscle mitochondria isolation

Characterization of age-related changes to mitochondria has been complicated by the fact that two distinct populations of mitochondria exist. Subsarcolemmal mitochondria (SSM) are located beneath the plasma membrane, while interfibrillar mitochondria (IFM) are found in parallel rows between the myofibrils [61]. Gastrocnemius muscle was quickly removed and tissue weights were recorded. IFM and SSM were isolated as described previously [18]. Briefly, after removing fat and tendons, gastrocnemius muscle (∼2.3 g) was minced in ice-cold isolation buffer (75 mM KCl, 150 mM sucrose, 1 mM EGTA, 5 mM MgCl_2_, 50 mM Tris-HCl, 1 mM KH_2_PO4, and 0.4% fatty acid-free BSA, pH 7.4 at 4°C), followed by homogenization in 10 ml buffer per gram of tissue on ice, using five full strokes with a mechanically driven Potter-Elvehjem glass-Teflon homogenizer. Homogenates were centrifuged (800×*g*, 10 min, 4°C) to separate large organelles and filaments containing IFM. The supernatant containing SSM was filtered through synthetic cheese cloth to avoid contamination with cell debris and was centrifuged (8,000×*g*, 10 min, 4°C) to obtain the SSM fraction. The initial IFM-containing pellet was immediately resuspended in 10 ml isolation buffer. IFM were released from myofibrils via incubation on ice with freshly prepared protease (0.5 U/g tissue; Sigma-Aldrich, St. Louis, MO) for 1 min with agitation. After five strokes of homogenization, large organelles and nuclei were removed by centrifugation (800×*g*, 10 min, 4°C). The supernatant containing IFM was filtered through cheese cloth and centrifuged (8,000×*g*, 10 min, 4°C) to collect the IFM pellet. The IFM and SSM pellets were gently resuspended in BSA-free buffer, centrifuged twice and kept on ice for the mitochondrial calcium retention capacity assay.

### Mitochondrial calcium retention capacity measurement

The maximum amount of Ca^2+^ required for mitochondrial PTP opening was measured on freshly isolated IFM and SSM using the fluorescent probe calcium green-5N (Molecular Probes, Eugene, OR) as described previously [18], [61]. IFM (0.2 mg/ml) and SSM (0.5 mg/ml) were incubated with 250 µl of mitochondrial PTP working buffer (250 mM sucrose, 10 mM KH_2_PO_4_ and 25 mM Tris-HCl, pH 7.4 at 37°C). The reaction was kept at 37°C and energized with 5 µl of substrate (0.5 M glutamate and 0.25 M malate). A Synergy HT multidetection microplate reader (Bio-TeK Instruments, Vinooski, VT) with automatic injectors was used to inject 1.25 nmol CaCl_2_ into each well, with a 1-min interval between injections. During this time, extra-mitochondrial Ca^2+^ pulses were recorded in the presence of 1 µM calcium green-5N, with excitation and emission wave lengths set at 506 and 532 nm, respectively. Ca^2+^ injection was continued until mitochondrial PTP completely opened. In a parallel assay, 0.5 µM of cyclosporin A (Sigma, St. Louis, MO) was used to confirm the release of Ca^2+^ in response to mitochondrial PTP opening [62].

### Mitochondrial protein carbonyl assay

Mitochondrial protein carbonyls were measured using a commercial ELISA (Biocell PC Test, Papatoetoe, New Zealand), following the manufacturer's instructions. In each well, 10 µg of mitochondrial protein was loaded and the final absorbance was read at 450 nm using a Spectramax 340 microplate spectrophotometer (Molecular Device, Sunnyvale, CA). The protein carbonyl levels (nmol/mg) were calibrated against oxidized protein standards provided.

### Measurement of RNA and DNA oxidation using HPLC-ECD

Oxidative RNA and DNA was quantified as 8-oxo-7,8-dihydroguanosine/10^6^ guanosine and 8-oxo-7,8-2′-deoxyguanosine/10^6^ deoxyguanosine using a HPLC-ECD method [2], [56], [63]. This procedure is based on high-salt nucleic acid release from proteins, followed by removal of proteins and fats by organic solvents at neutral pH, all in the presence of the metal chelator deferoxamine mesylate (DFOM) at 0°C. Muscle pieces were thawed, stripped for tendons on ice, weighed (∼150 mg), minced, and homogenized on slush ice using a glass-glass Duall homogenizer in 2 ml (1∶10 w∶v) buffer (3 M guanidine thiocyanate (GTC), 0.2% (w/v) N-lauroylsarcosinate, 20 mM Tris, pH 7.5) containing 10 mM freshly prepared DFOM. After transferring the solution into phase-lock gel (PLG) tubes, an equal volume of phenol/chloroform/isoamyl alcohol (25∶24∶1, pH 6.7) was added and the samples were immediately vortexed, followed by a 10 min vortexing period at 0°C to completely release nucleic acids as previously described [63]. After centrifugation (4,500×*g*, 5 min, 0°C), the aqueous phase was transferred into a new PLG tube and extracted with an equal volume of chloroform/isoamyl alcohol (24∶1). The samples were hand-shaken, centrifuged, and the aqueous phase was collected and nucleic acids were precipitated by addition of an equal amount of isopropanol. After centrifugation (10,000×*g*, 10 min, 0°C), nucleic acids were washed with 70% (v/v) ethanol, dried, dissolved in DNAse and RNase-free water containing 30 µM DFOM, and hydrolyzed using 4 U nuclease P_1_ and 5 U alkaline phosphatase in buffer (30 mM sodium acetate, 20 µM ZnCl_2_, pH 5.3) at 50°C for 60 min. After filtration, the samples were analyzed by high-performance liquid chromatography coupled to electrochemical and UV detection (HPLC-ECD/UV).

### Total urine antioxidant power

Total urine antioxidant power was measured by using NEOGEN calorimetric assay kits (Neogen Corporation, Lexington, KY). The assay uses antioxidants provided by the urine samples or uric acid standards to reduce Cu(II) to Cu(I). The reduced form of copper selectively reacts with the chromogenic reagent to form a complex, which is stable and has an absorption maximum at 450 nm. Total urine antioxidant power was calibrated against uric acid standards provided with kits. Protein concentration was assayed with the Bradford method using bovine serum albumin as the standard [64]. Data were expressed as µmol uric acid equivalents per mg urine protein.

### Ferric reducing antioxidant power in plasma and muscle tissue

The ferric reducing antioxidant power (FRAP) assay was performed according to Benzie and Strain [65]. The principle of the assay is based on the reduction of the Fe^3+^-2,4,6-tripyridyl-S-triazine complex to the ferrous form (Fe^2+^). The antioxidant activity of gastrocnemius muscle homogenate (1∶10 w∶v) or plasma was measured by monitoring the change in absorption at 593 nm. Acetate buffer (0.3 M, pH 3.6) was prepared by dissolving 3.1 g C_2_H_3_O_2_Na•3H_2_O and 16 ml acetic acid in 1 liter double-distilled water. 2,4,6-Tripyridyl-S-triazine solution was prepared by dissolving 10 mmol in 1 liter 40 mM HCl solution. Ferric solution (20 mmol/l) was prepared using FeCl_3_•6H_2_O. The final working FRAP reagent was prepared freshly by mixing acetate buffer, 2,4,6-tripyridyl- S-triazine and ferric solutions at a ratio of 10∶1∶1 (by vol.). In brief, 200 µl FRAP working reagent were incubated at 37°C in Spectramax 340 microplate spectrophotometer for 30 min. The reagent blank reading was recorded at 593 nm followed by adding 10 µl plasma or muscle homogenate. The absorbance was taken at 20 min when the reading was constant. The difference in absorbance between the tested sample and the blank reading was calculated and the data were calibrated with ferrous standards and expressed as mM.

### Statistical analysis

Results are expressed as means ± SEM. Statistical analyses were performed using GraphPad Prism Version 4.0 (GraphPad Software, San Diego, CA). For outcome measures, effects were assessed using a one-way ANOVA with age (8, 21, and 29) as a factor in control groups and a two-way ANOVA with treatment (Eufortyn® and control) and age (21 and 29) as factors. Where appropriate, post-hoc effects were analyzed using a Tukey's multiple comparison test. Significance was set at *p*<0.05. There was no statistical difference unless indicated otherwise.

## Results

### Food intake

Food intake increased with age (age, *p*<0.05, one-way ANOVA). However, there was no significant difference in food intake between Eufortyn® treated rats and their age-matched counterparts (**Table 3**).

**Table 3:** Baseline chow intake, body and muscle weights for male F344BNF1 rats.

Age (months)	8	21	21	29	29
Group	Control	Control	Eufortyn®	Control	Eufortyn®
BW (g)	405±12 ^a^	518±13 ^b^	523±10^ b^	518±19 ^b^	556±18 ^b^
N	7	14	13	7	7
Food intake (g/day)	16.7±0.7 ^a^	18.9±0.4 ^ab^	19.0±0.5^ ab^	19.7±1.1 ^b^	20.8±0.8^ b^
Gastrocnemius/BW (mg/g)	5.31±0.06 ^c^	4.11±0.12 ^b^	4.00±0.14^ b^	3.10±0.11 ^a^	3.03±0.11^ a^
Plantaris/BW (mg/g)	1.02±0.02 ^c^	0.89±0.03 ^b^	0.89±0.02^ b^	0.70±0.06 ^a^	0.68±0.04^ a^
Quadriceps/BW (mg/g)	7.80±0.36 ^c^	6.44±0.18 ^b^	6.21±0.19 ^b^	5.30±0.26 ^a^	5.26±0.18^ a^
Soleus/BW (mg/g)	0.41±0.01 ^b^	0.38±0.02 ^b^	0.36±0.01^ b^	0.30±0.02 ^a^	0.31±0.01^ a^
EDL/BW (mg/g)	0.44±0.01 ^c^	0.37±0.01 ^b^	0.37±0.01^ b^	0.29±0.02 ^a^	0.30±0.00^ a^

Values are means ± SEM (n = 7−14).

a,b,c Different letters are significantly different from each other (*p*<0.05 by Tukey's Multiple Comparison Test). Eufortyn® did not affect body and muscle weights, as well as food intake at 21 and 29 months of age. The muscle weight is reported as the average weight of muscles from two legs. Abbreviations: BW, body weight; EDL: extensor digitorum longus muscle.

### Body weight

Body weight was significantly different between 8 and 21 months of age (*p*<0.05, Tukey's multiple comparison test), but did not show further change between 21 and 29 months of age in control animals (**Table 3**). Eufortyn® supplement did not affect body weight in rats at both 21 and 29 months of age as compared with their age-matched control groups.

### Muscle mass

Across the ages tested, there were differences in muscle mass as measured in the gastrocnemius, plantaris, quadriceps, soleus and extensor digitorum longus (EDL) muscles (age, *p*<0.05, one-way ANOVA) (**Table 3**). Post-hoc analysis indicated that quadriceps, gastrocnemius and plantaris muscle groups, the major antigravity extensor muscles of the hindlimb, weighed significantly less in 29-month-old rats than in 21-month-old rats (*p*<0.05, Tukey's multiple comparison test). In addition, the mass of EDL (fast-twitch) and soleus (slow-twitch) muscles showed significant age-associated reductions. Overall, Eufortyn® supplementation did not affect muscle mass in rats at both 21 and 29 months of age as compared with their age-matched control groups.

### Grip strength

**Figure 1:**
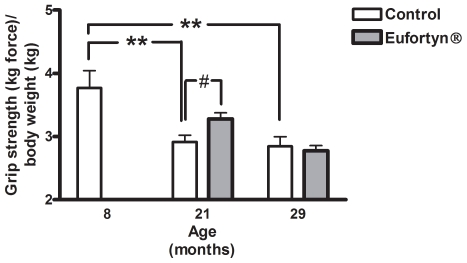
Eufortyn® supplementation affected grip strength in rats at 21 months of age. In the control groups, there was a significant age-related decline in grip strength (age, *p*<0.01, one-way ANOVA). Post-hoc analysis showed a decline in 21- and 29-month-old rats (**p<0.01, Tukey's multiple comparison test) as compared to 8-month-old control cohort. There was a significant increase in grip strength in 21-month-old rats fed Eufortyn® as compared to their age-matched controls after 4 weeks of treatment (^#^ *p*<0.05, Tukey's multiple comparison test). Values are means ± SEM (n = 7−14).

In the control groups, there was a significant age-associated decline in grip strength (age, *p*<0.01, one-way ANOVA) with a 23% decrease at 21 months (*p*<0.01, Tukey's multiple comparison test) and a 24% decrease at 29 months (*p*<0.01, Tukey's multiple comparison test) as compared to 8-month-old rats (**Fig. 1**). There was a significant attenuation of declining grip strength (*p*<0.05, Tukey's multiple comparison test) in 21-month-old rats fed Eufortyn® as compared to age-matched controls. There was no difference in grip strength, however, between control and Eufortyn® groups at 29 months of age.

### Ca^2+^ retention capacity in muscle SSM and IFM

**Figure 2:**
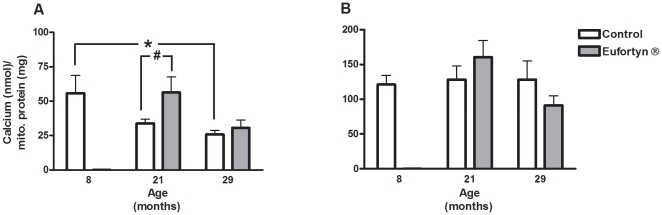
Eufortyn® significantly improved the calcium retention capacity in muscle subsarcolemmal mitochondria (SSM) in 21-month-old rats. (A) The maximum amount of Ca^2+^ required for mitochondrial PTP opening was measured on freshly isolated IFM and SSM. The calcium retention capacity of the SSM for control rats at 8, 21 and 29 months of age decreased significantly with age (age, *p*<0.05, one-way ANOVA). However, the decrease in the calcium uptake capacity in SSM of 21-month-old rats was significantly ameliorated by Eufortyn® supplementation (^#^ *p*<0.05, Tukey's multiple comparison test). (B) The calcium retention capacity of IFM for rats at 8, 21, and 29 months of age did not change over time and there was no statistical difference in the IFM calcium uptake capacity between control and treatment animals at 21 and 29 months of age. Values are means ± SEM (n = 7−14).

The maximum amount of Ca^2+^ required for mitochondrial PTP opening was measured on freshly isolated IFM and SSM. The calcium retention capacity of the SSM decreased significantly with age in control rats at 8, 21 and 29 months of age (age, *p*<0.05, one-way ANOVA) (**Fig. 2A**). No age-associated changes in the IFM, however, were observed in control groups (**Fig. 2B**). Eufortyn® supplement could significantly increase Ca^2+^ retention capacity of SSM in the muscle of 21-month-old rats as compared to their age-matched counterparts (*p*<0.05, Tukey's multiple comparison test). However, Eufortyn® did not affect Ca^2+^ retention capacity of SSM in the muscle of 29-month-old rats as well as that of IFM in the muscle of 21- and 29-month-old rats.

### Protein carbonyl levels in muscle SSM and IFM

**Figure 3:**
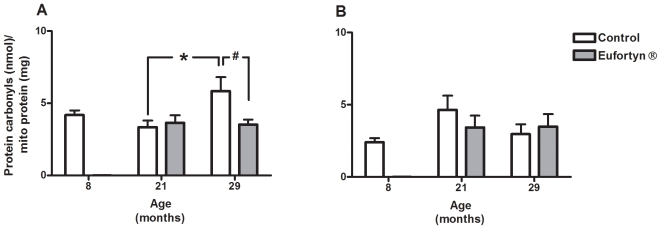
Eufortyn® supplementation significantly decreased the subsarcolemmal mitochondria (SSM) protein carbonyl concentration in 29-month-old rats as compared to the age-matched control cohort. (A) SSM protein carbonyl concentrations increased with age (age, *p*<0.05, one-way ANOVA). There was a significant difference in the SSM protein carbonyl concentration between 21- and 29-month-old control groups (*p<0.05, Tukey's multiple comparison test). Eufortyn® significantly ameliorated the protein carbonyl levels in SSM in 29-month-old rats (^#^ *p*<0.05, Tukey's multiple comparison test). (B) The protein carbonyl concentrations in IFM did not change over time for control animals. Moreover, there was no treatment effect or interaction. Values are means ± SEM (n = 5−10).

The levels of protein carbonyls significantly increased in SSM during the aging process in control cohorts (age, *p*<0.05, one-way ANOVA) (**Fig. 3A**). Although the protein carbonyl levels in the SSM of 21-month-old control rats did not show any increase in comparison with that of 8-month-old control rats, SSM protein carbonyl levels significantly increased between 21- and 29-month-old control rats (*p*<0.05, Tukey's multiple comparison test). Eufortyn® could significantly lower the increase of protein oxidation in SSM in the muscle of 29-month-old rats (*p*<0.05, Tukey's multiple comparison test). Two-way ANOVA analysis indicated that there was a significant interaction of main effects (*p*<0.05) and it showed that the effects of Eufortyn® treatment depended on the age of the rats. Conversely, age and Eufortyn® supplementation did not affect the protein carbonyl levels in IFM (**Fig. 3B**).

### Nucleic acid oxidative damage in gastrocnemius muscle

**Figure 4:**
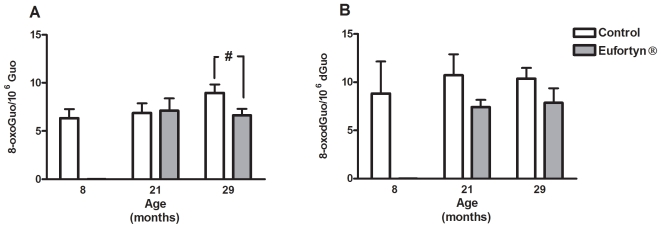
RNA and DNA oxidative damage in gastrocnemius muscle. (A) Post-hoc analysis showed that there is a tendency for greater level of RNA oxidation in 29-month-old rats compared to 8-month-old control (*p* = 0.09, Tukey's multiple comparison test). In addition, Eufortyn® significantly decreases RNA oxidative damage in 29-month-old rats (^#^ *p*<0.05, Tukey's multiple comparison test). (B) DNA oxidative damage did not change over time for control animals. Values are means ± SEM (n = 7−8).

There was a trend (*p* = 0.09, Tukey's multiple comparison test) for greater level of RNA oxidation in gastrocnemius muscle of 29-month-old control rats as compared to 8-month-old controls (**Fig. 4A**). In contrast, the RNA oxidation levels remained fairly constant with age within Eufortyn® groups and there was a significant decrease (*p*<0.05, Tukey's multiple comparison test) in muscle RNA oxidation in 29-month-old rats fed Eufortyn® in comparison to that of their age-matched counterparts. In control groups, the DNA oxidation levels did not change significantly over the course of aging (**Fig. 4B**). Two-way ANOVA indicated a tendency for DNA oxidation levels to be lower in rats fed Eufortyn® compared to control rats (treatment, *p* = 0.07).

### Total urine antioxidant power

The total urine antioxidant power in control groups decreased with age (age, *p*<0.0001, one-way ANOVA) (**Table 4**). Post-hoc analysis indicated that total urine antioxidant power in 29-month-old rats was remarkably lower than that in 8- and 21-month-old rats (*p*<0.001, Tukey's multiple comparison test). In contrast, Eufortyn® significantly increased the total urine antioxidant power in 29-month-old rats as compared to age-matched control (*p*<0.001, Tukey's multiple comparison test) (**Table 4**).

**Table 4:** Total urine antioxidant power for male F344BNF1 rats.

Age (months)	8	21	21	29	29
Group	Control	Control	Eufortyn®	Control	Eufortyn®
µmol uric acid equivalent per mg protein	3.56±0.07 ^b^	3.16±0.15 ^b^	3.53±0.09 ^b^	1.57±0.17 ^a^	3.86±0.10 ^b^

Values are means ± SEM (n = 3−7).

a,b Different letters are significantly different from each other (*p*<0.001 by Tukey's Multiple Comparison Test).

### Ferric reducing antioxidant power in muscle tissue and plasma

In agreement with the total antioxidant results in the urine, there was a 35% increase in the FRAP of gastrocnemius muscle in 29-month-old rats fed Eufortyn® in comparison with their age-matched counterparts (**Table 5**). Moreover, Eufortyn® supplementation increased plasma FRAP by 15% in 29-month-old rats (**Table 6**), indicating an augmented antioxidant power in tissues and cells. However, there was no statistical difference in muscle and plasma FRAP due to the low sample size. Most sample specimens were utilized, for example we only had 3 samples left in 29-month-old control cohort (**Table 5** **and** **6**).

**Table 5:** Ferric reducing antioxidant power (FRAP) of gastrocnemius muscle homogenate in 8- and 29-month-old control rats and in 29-month-old Eufortyn® treated rats.

Age (months)	8	29	29
Group	Control	Control	Eufortyn®
N	4	3	6
FRAP value (mM)	0.228±0.020	0.205±0.002	0.276±0.036

Values are means ± SEM (n = 3−6). Note the small sample size in the 29-month control group, which limits the statistical power of this particular analysis. This was due to the limited remaining sample availability (See results).

**Table 6:** Ferric reducing antioxidant power (FRAP) of plasma in 8-month-old control rats and in 21- and 29-month-old rats fed either control or Eufortyn® pellets.

Age (months)	8	21	21	29	29
Group	Control	Control	Eufortyn®	Control	Eufortyn®
N	6	6	6	3	4
FRAP value (mM)	0.393±0.008	0.395±0.009	0.408±0.025	0.314±0.024	0.360±0.004

Values are means ± SEM (n = 3−6). Note the small sample size in the 29-month control group, which limits the statistical power of this particular analysis. This was due to the limited remaining sample availability (See results).

## Discussion

The purpose of this study was to assess the effects of Eufortyn® supplementation on oxidative stress, mitochondrial function and physical performance. We have demonstrated that Eufortyn® significantly attenuates declining physical performance and improves Ca^2+^ retention capacity of SSM in 21-month-old rats. Moreover, the age-related increase in cellular RNA oxidation and protein oxidation in SSM was significantly ameliorated by Eufortyn®. These findings suggest that Eufortyn® may improve homeostatic regulation of cellular and mitochondrial oxidative stress, mitochondrial function and physical performance if initiated at early middle age.

### Functional aging and grip strength

The consequences of the aging process in skeletal muscles are characterized by a decline in physiological function and a loss of muscle strength, a condition incapable of performing strenuous physical work [66]. The term “functional age”, therefore, is a reliable index of the most vulnerable subset of older individuals [67], [68]. The primary outcome variable of this study was grip strength. In both humans and rodents, grip strength is one of most representative measurements in functional aging [69], [70]. Our previous studies have shown that aged rats with advanced muscle atrophy exhibited reduced grip strength [55], [56]. In the present study, muscle mass and grip strength decreased significantly with age in control groups (**Table 3**). Eufortyn® supplementation significantly ameliorated the decline in grip strength in 21-month-old rats, however, did not have a significant effect when initiated in 29-month-old rats. This suggests there may be a critical time period during which this nutritional combination is effective and they may no longer be beneficial after a threshold is reached. Another possibility is that the time period of Eufortyn® supplementation (4 weeks while grip strength was measured and total of 6 weeks in this study) and the dosage used were not sufficient to achieve this goal in late middle aged animals.

### Mitochondrial PTP opening in the gastrocnemius muscle

Mitochondrial oxidative stress and apoptotic cell death are being increasingly recognized as fundamental driving mechanisms of aging, especially in post-mitotic tissues [1], [66], [71]. The switch to a cell death process can be mediated by opening of the mitochondrial PTP, causing collapse of the membrane potential and swelling [19]. Therefore, inhibition of pore opening is an effective and promising strategy to modulate the aging process.

In the present study, gastrocnemius muscles were selected for mitochondrial isolation and function measurement because (1) they are one of the major antigravity extensor muscles of the hindlimb; (2) the substantial amount of tissue allowed us to perform the analysis; (3) forelimb and hindlimb grip force is positively correlated in rodents [59]. Our current study clearly shows that Eufortyn® efficiently mitigated the susceptibility of SSM to Ca^2+^-induced mitochondrial PTP opening and improved SSM function in 21-month-old rats. Although the increase in forelimb muscle force as assessed by grip strength cannot be directly linked to the improved muscle mitochondrial calcium handling capacity observed in the hindlimb muscle, it is remarkable that both grip strength and mitochondrial Ca^2+^ handling capacity increased by Eufortyn® supplementation in 21-month-old rats.

Consistent with our previous study [18], we also found that there was a significant age-associated decrease in mitochondrial Ca^2+^ retention capacity in SSM, but not in IFM. Post-hoc analysis indicated that the SSM isolated from late middle aged rat muscle have a significant lower Ca^2+^ retention capacity than those from young rat muscle, whereas the IFM Ca^2+^ retention capacity remained fairly constant within all age cohorts. Indeed, various studies have shown that the IFM and SSM differ in intracellular localization and function [72]–[75]. For instance, Adhihetty *et al.* have measured how fast the PTP in IFM and SSM opened in response to excessive Ca^2+^ and oxidative stress and they found that IFM exhibited a greater “Time to Vmax” compared to SSM, indicating that IFM were more resistant to Ca^2+^-induced PTP opening than SSM [72]. This is in agreement with our results, suggesting that the two subpopulations differ in function measurements.

### Oxidative stress in the gastrocnemius muscle

Protein carbonyl content is one of the most widely used oxidative markers and increased levels of protein carbonyls have been reported in patients with diabetes [76], breast cancer [77] and aging [78]. Recently, age-related increase in total mitochondrial protein carbonyl levels in skeletal muscles has been reported in rats, showing that carbonyl levels in skeletal muscles are associated with reactive oxygen species produced in mitochondria [79]. Supportively, our results indicate that the SSM protein carbonyl levels was significantly elevated in 29-month-old rat gastrocnemius muscles and Eufortyn® supplementation remarkably ameliorated the increase in SSM protein carbonyl levels. Recently, Barreiro and Hussain have fully documented the association between elevated protein carbonylation and depressed skeletal muscle performance [80]. There is evidence of skeletal muscle immobilization and elevated protein carbonylation in rodents undergoing mechanical ventilation [81], [82]. The observation that administration of antioxidants significantly mitigated mechanical ventilation-induced contractile dysfunction and protein carbonylation further underlines the importance of antioxidant defense [83], [84].

Accordingly, oxidative damage to DNA and RNA in the gastrocnemius muscle was assessed as their oxidation products simultaneously. We found that muscle RNA oxidative damage product has the tendency to increase at 29 month of age. The functional consequences of increased RNA oxidative damage in skeletal muscles could be serious and even causative to muscle contraction and atrophy [2], [85]. Therefore, the decline in grip force in 29-month-old control rats may be related to the increased SSM protein and RNA oxidation levels in the gastrocnemius muscle.

### Total urine antioxidant power and plasma ferric reducing antioxidant power

The decrease in total antioxidant power in plasma or body fluids has been implicated in the aging process and a number of diseases [86]–[88]. For instance, Leeuwenburgh *et al*. reported an age-related increase in urinary oxidized amino acids in rats and antioxidant supplementation partly attenuated the increase [89]. Thus, total antioxidant capacity measurements can provide the integrated characteristics of endogenous redox potential and also give a further insight into the effectiveness of dietary modulation *in vivo* on redox status [90], [91]. We have confirmed that there was a significant decrease in total urine antioxidant power in late middle aged rats; however, the decrease was completely reserved by Eufortyn®. The data imply that Eufortyn® is effective in exerting its antioxidant capacity and modulating *in vivo* redox status, not only in the skeletal muscle, but also in the metabolic system. Moreover, the changes in total urine antioxidant power as well as in plasma and muscle FRAP after supplementation may provide information on the absorption and bioavailability of nutritional compounds, suggesting that Eufortyn® can be effectively absorbed and its metabolites excreted in the urine.

Eufortyn®, by virtue of its antioxidant capacity, significantly decreased the SSM protein carbonyl levels and muscle RNA oxidative damage in 29-month-old rats. Consistently, the decrease of total urine antioxidant power in 29-month-old rats was mitigated by Eufortyn® supplementation, suggestion that Eufortyn® exerts potent antioxidant activity and was distributed throughout the circulation. However, it failed to completely reverse the loss of grip strength decrease in 29-month-old rats, suggesting that other causal factors are likely to be involved and more studies are warranted to investigate the effective dosage and supplementation time period of Eufortyn® in late middle aged animals.

### Conclusions

The current study provides striking results concerning the potential benefits of Eufortyn® used to combat age-related mitochondrial dysfunction. Gastrocnemius muscle mitochondrial function and grip strength were improved in 21-month-old rats fed Eufortyn® compared with their age-matched counterparts. In addition, muscle mitochondrial protein oxidation (carbonyl formation), oxidative stress to RNA, and the age-associated decline of total urine antioxidant power were remarkably attenuated by Eufortyn® supplementation in 29-month-old rats. Our current study suggests that Eufortyn® is a potent antioxidant and has the potential to improve health span. Future clinical studies should explore the potential benefits of Eufortyn® on physical function, clinical outcomes, as well as its ability to reduce oxidative stress levels, in middle-aged and older adults.
